# Examination of Acceptability, Feasibility, and Iatrogenic Effects of Ecological Momentary Assessment (EMA) of Suicidal Ideation

**DOI:** 10.1177/10731911231216053

**Published:** 2023-12-14

**Authors:** L. M. M. Kivelä, F. Fiß, W. van der Does, N. Antypa

**Affiliations:** 1 Leiden University, The Netherlands; 2 Leiden University Treatment and Expertise Centre (LUBEC), The Netherlands

**Keywords:** suicide, reactivity, ambulatory assessment, experience sampling method

## Abstract

Ecological momentary assessment (EMA) can be used to examine the dynamics of suicidal ideation in daily life. While the general acceptability and feasibility of EMA in suicide research has been established, further examination of potential iatrogenic effects (i.e., negative reactivity) and identifying those more likely to react negatively is needed. Participants (*N* = 82) with current suicidal ideation completed 21 days of EMA (4×/day) and filled in *M* = 78% (*Med* = 84%) of the EMA. No positive or negative affect reactivity was observed in EMA ratings over the study period. Retrospectively, most participants rated their experience as positive (69%); 22% indicated mood worsening, and 18% suicidal ideation reactivity. Those with more borderline personality traits, posttraumatic stress disorder (PTSD), and higher depressive, anxiety, and suicidal ideation symptoms, were more likely to report iatrogenic effects. In conclusion, while high compliance rates and lack of affect reactivity during EMA indicate that EMA is well tolerated in suicide research, a minority of participants may report subjective mood effects in retrospect.

Ecological momentary assessment (EMA) is an emerging methodology in suicide research (Davidson et al., 2017). EMA encompasses data collection methods where participants are repeatedly prompted to report on their experiences, as part of their normal daily lives and in real time, using electronic devices (Shiffman et al., 2008). Data may thus be collected in a way that increases ecological validity, minimizes recall bias, and enhances the temporal granularity of the information collected. Recent reviews (Gee et al., 2020; Kivelä et al., 2022; Sedano-Capdevila et al., 2021) have demonstrated that EMA can be used for the real-time assessment of suicidal ideation and its associated momentary risk factors. EMA allows for the assessment of more dynamic characteristics of suicidal ideation, such as hourly and daily fluctuations in the intensity of ideation, as well as risk factors that may be time- or context-dependent (Myin-Germeys et al., 2018). While the use of EMA in suicide research is growing rapidly, few studies so far have directly examined the feasibility and acceptability of EMA in suicide research, especially in terms of potential iatrogenic effects (i.e., negative reactivity to EMA). More data are also needed on the subjective experience of participants in such studies. Specifically, there may be concern about the burden imposed on already vulnerable populations, as well as the potentially harmful effects of repeated assessments of suicidal ideation (Bos, 2021).

The possible iatrogenic effects of suicide assessments have been a long-time concern of both clinicians and researchers. A 2009 survey of medical ethics committee members revealed that 65% believed that participating in suicide-related research would be detrimental to patients (Lakeman & FitzGerald, 2009). However, the consensus from the general literature indicates that inquiring people about their suicidal ideation, even when done repeatedly or intensively, does not increase suicidal ideation, or trigger suicidal or self-harm behavior (Bender et al., 2019; Gould et al., 2005; Hom et al., 2018; Schatten et al., 2022; Smith et al., 2010). Some studies have shown that such assessments may even serve to lessen ideation and associated distress: for example, in a study involving interview and questionnaire measures, as well as exposure to suicide-related stimuli as part of an emotional picture processing task, participants reported reductions in suicidal ideation at 1-month follow-up (Schatten et al., 2022). A 2018 review and meta-analysis of 13 studies examining iatrogenic effects of suicide assessments also concluded that no significant negative outcomes resulted from participation (DeCou & Schumann, 2018). However, these findings may not extend to study designs where measures of suicidal ideation may be repeated up to a hundred times over the span of days and weeks. Another concern therefore regards the compliance of patients to EMA designs, whether influenced by negative reactivity to the assessments, or the general burden of such intensive research designs.

Studies to date appear to support the feasibility, acceptability, and safety of EMA in suicide research. In the first study examining the feasibility of EMA-based suicidal ideation assessments, Husky et al. (2014) found study acceptability (i.e., agreement to participate) to be higher among recent suicide attempters (88%) than healthy controls (77%), although compliance among cases (74%) was lower than controls (86%). Subsequent studies have largely supported these early findings: based on a review of 23 EMA studies examining suicidal ideation, median acceptability was 77%, and compliance (i.e., average response rate) was 70% (Kivelä et al., 2022). Excellent retention rates were also reported (*Med* = 94%) (Kivelä et al., 2022). These numbers mirror those derived from EMA studies in other clinical populations (Johnson et al., 2009).

However, fewer studies have directly examined iatrogenic effects of EMA. Most studies have concluded on the acceptability of EMA based on objective indices, such as high retention and compliance rates. Husky and colleagues (2014) also examined reactive effects, and found that the intensity and frequency of negative affect and suicidal ideation did not increase as a function of study duration, indicating no negative reactivity to repeated assessments. However, this study only lasted 7 days, while EMA studies may frequently use weeks-to-months long assessments (range in prior EMA studies on suicidal ideation 4–60 days; Kivelä et al., 2022). Another study comparing a 14-day EMA protocol on suicidal ideation to a control protocol (14 days of EMA on negative psychological experiences with no suicide-related items) found no differences in the occurrence of suicidal ideation, attempts or self-harm between the two groups; these findings were replicated both among clinical cases (borderline personality disorder) and controls (Law et al., 2015). Furthermore, the effects of frequency of EMA on suicidal ideation severity were examined in a sample of 101 adults with past-week active suicidal ideation; no negative effects were observed (Coppersmith et al., 2022). However, more nuanced effects may occur. For example, while Husky and colleagues (2014) found no effects on the key outcomes of negative affect and suicidal ideation, decreases in both positive affect *and* hopelessness were observed. Consequently, both potential negative as well as positive reactive effects to EMA need to be further evaluated.

With regard to participants’ subjective experience with EMA studies, most participants have rated their experiences as “neutral-to-positive” based on two studies, one in a sample of 34 adolescents who completed once-daily EMA for 21 days (Czyz et al., 2018), and another in a sample of 237 high-risk adults from the community who completed EMA six times per day over 14 days (Rogers, 2021). Participants in both studies predominantly indicated that they would participate in similar research again (Czyz et al., 2018; Rogers, 2021). However, subsets of participants reported having experienced the EMA protocol as stressful and/or burdensome (16%) (Forkmann et al., 2018), occasionally distressing and/or triggering bad thoughts (9%) (Rogers, 2021), or having made them feel worse (3%) (Czyz et al., 2018). Notably, to the best of our knowledge, no previous study has examined the characteristics of participants who are more likely to report negative reactivity from EMA assessments. Consequently, predictors of iatrogenic effects warrant further examination.

The aim of this study was to enrich the current literature on the acceptability, feasibility, and safety of EMA in suicide research by presenting data from the SAFE study, a longitudinal cohort study in individuals with current suicidal ideation, in which mobile-phone-based EMA (4 ×/day) was administered over 3 weeks. Specifically, we aimed to replicate prior findings indicating that EMA of suicidal ideation does not result in systematic iatrogenic effects on suicide outcomes (Coppersmith et al., 2022; Husky et al., 2014; Law et al., 2015). Furthermore, we comprehensively assessed participants’ subjective experiences as relating to study participation (extending on Czyz et al., 2018; Forkmann et al., 2018; Rogers, 2021). While prior studies have indicated no systematic reactivity with EMA on suicidal ideation or behavior specifically (Coppersmith et al., 2022; Husky et al., 2014; Law et al., 2015), reactivity on other outcomes (such as reduced positive affect; Husky et al., 2014) has been reported and warrants further examination. We therefore aimed to further replicate the prior findings indicating that EMA of suicidal ideation does not result in suicidal reactivity, and explore effects on other (positive/negative) affect outcomes. Furthermore, identifying (groups of) participants who might be more at risk to react negatively is of both research and clinical value because some participants do self-report iatrogenic effects (Czyz et al., 2018; Rogers, 2021), indicating the need to better characterize this subgroup at risk. In sum, while the application of EMA in suicide research is ever-growing, only a few studies have reported on reactive effects, and participant characteristics associated with an increased likelihood of reporting iatrogenic effects have not previously been examined. This information is important to ensure that the field progresses in a safe manner. To this extent, we examined (a) acceptability and feasibility (incl. agreement to participate, attrition, compliance), (b) predictors of compliance (i.e., how baseline characteristics affect response rates), and (c) iatrogenic effects (i.e., whether systematic changes could be observed in participants’ affect and/or suicidal ideation ratings over the study period, and which participants were most likely to be subject to reactivity). Finally, we explored participant feedback given at the end of the 3-week EMA period on their subjective experience with the assessments.

## Methods

### Participants

Eligible participants were 18 years or older with a recent (past year) history of a suicide attempt and/or active suicidal ideation, based on a reduced version of the Columbia Suicide Severity Rating Scale (CSSRS) (Posner et al., 2011) comprised of the first five questions, with cutoff scores of ≥3, or ≥2 if symptoms were present in the past 2 months. Participants had a sufficient proficiency in written and spoken English and/or Dutch; possessed an Android or iOS compatible smartphone; and were registered with a local (Dutch) general practitioner (GP). Exclusion criteria included a current diagnosis of bipolar disorder, a psychotic disorder, or (severe) substance dependence (based on *Diagnostic and Statistical Manual of Mental Disorders* [5th ed.; *DSM-5*; American Psychiatric Association, 2013] criteria).

### Instruments

#### Intake Interview

Data on participants’ sociodemographic characteristics and medical and psychiatric history (incl. medications) were collected through a custom semistructured interview. A reduced version of the CSSRS was used to assess the participants’ recent (past year) history of suicidal ideation; additional questions were included on lifetime history of suicide attempts. The MINI Neuropsychiatric interview (version 5.0) (Sheehan et al., 1998) and the Structured Clinical Interview for *DSM-5* Personality Disorders subscale for Borderline Personality Disorder (SCID-PD-BPD) (First, 2015) were used to establish current diagnoses.

#### Ecological Momentary Assessment

Each EMA assessment included the same core set of questions, with additional questions on sleep parameters included as part of the morning assessment, and questions about napping included as part of the evening assessment. The full set of EMA questions, item formulation and rating scales can be found in Supplementary Material. The core set of questions covered the participants’ current: (a) location, social company, and activity, (b) affect (happiness, calmness, sadness, anxiety, anger, guilt, and shame), (c) cognitions (hopelessness, loneliness, burdensomeness, and optimism), (d) suicidal ideation (passive and active ideation, acquired capability), (e) impactful events (type and stressfulness of positive and negative impactful events), (f) coping (use of coping strategies), and (g) substance use (medication, alcohol, and recreational drugs). Morning assessment of the previous night’s sleep included questions about the participants subjective sleep quality, timing of sleep, and experience of nighttime awakenings and nightmares; evening assessments inquired about napping during the day. Participants filled in 4×/day EMA over the first 20 days, and a final morning assessment on Day 21, resulting in a total of 81 scheduled entries. Additional data collected by the EMA app included response time (i.e., time from alert to response) and completion time (i.e., time to complete EMA once opened). EMA items used in the present analyses included suicidal ideation (mean of the three EMA items on desire to live, desire to die, and suicidal thoughts; *n.b.* desire to live was reverse coded prior to calculating the mean score), positive affect (mean of the EMA items on happiness and calmness) and negative affect (mean of the EMA items on sadness, anxiety, anger, guilt and shame). Descriptives of the study variables are presented in Table 1.

**Table 1.** Within-Person Descriptive Statistics of Study Variables.

Variable	M	*SD*	Range	ICC	RMSSD
Suicidal ideation (mean)	3.04	1.97	0–9	0.71	1.18
Desire to live	4.28	2.25	0–9	0.69	1.50
Desire to die	3.09	2.59	0–10	0.70	1.58
Suicidal thoughts	1.57	1.79	0–8	0.53	1.29
Positive affect (mean)	5.13	1.29	2–8	0.43	1.54
Happy	4.93	1.52	0–8	0.46	1.68
Calm	5.33	1.25	2–9	0.30	2.04
Negative affect (mean)	2.92	1.62	0–7	0.61	1.27
Sad	3.54	1.72	0–7	0.41	2.11
Anxious	3.59	1.80	0–8	0.44	2.14
Angry	1.87	1.48	0–6	0.38	1.88
Guilty	2.84	2.30	0–9	0.61	1.79
Ashamed	2.76	2.44	0–10	0.65	1.67

*Note.* M = mean; *SD* = standard deviation; ICC = intraclass correlation; RMSSD = root mean square of successive differences; based on scheduled entries *k* = 5,196; ‘desire to live’ is reverse coded i.e., higher scores reflect less desire to live.

#### Questionnaires

At baseline, participants filled in additional state and trait measures. The Beck Scale for Suicide Ideation (BSSI) (Beck et al., 1979) is a 21-item measure of current (past week) suicidal ideation. Cronbach’s alpha in our sample was .91. The Beck Depression Inventory (BDI-I) (Beck, 1961) is a 21-item measure of current (past week) depressive symptoms (Cronbach’s alpha = .85). The Hamilton Anxiety and Depression Scale—Anxiety Subscale (HADS-A) (Zigmond & Snaith, 1983) is a 7-item measure of current (past week) anxiety symptoms (Cronbach’s alpha = .65). The Insomnia Severity Index (ISI) (Bastien, 2001) is a 7-item measure of sleep complaints experienced in the previous 2 weeks (Cronbach’s alpha = .79). The Quality of Life Enjoyment and Satisfaction Questionnaire—Short Form (Q-LES-Q-SF) (Endicott et al., 1993) is a 16-item measure assessing current (past week) life satisfaction with regard to relationships, work, and health (Cronbach’s alpha = .85). The Leiden Index of Depression Sensitivity—Revised (LEIDS-R) (Solis et al., 2017) is a 34-item measure on the propensity to cognitive reactivity (Cronbach’s alpha = .85). The State-Trait Anger Expression Inventory (STAXI) (Zigmond & Snaith, 1983) is a 44-item measure on state and trait anger (expression); in this study, we used the 10-item trait subscale (Cronbach’s alpha = .84). Finally, the Personality Assessment Inventory—Borderline Scale (PAI-BOR) (Morey, 1991) is a 24-item measure of borderline personality traits (Cronbach’s alpha = .83). The same questionnaires were repeated after the 21-day EMA period (apart from the LEIDS-R, STAXI, and PAI-BOR which are trait measures and were not expected to change within the study period); in addition, participants also filled in a custom questionnaire on their experience with the EMA procedure (see Supplementary Material).

### Procedure

#### Recruitment

Participants for the study were recruited through fliers distributed in the community and on social media, as well as the Leiden University Medical Center (LUMC) Department of Psychiatry, Leiden University Treatment and Expertise Center (LUBEC), and other collaborating treatment centers in the area of Leiden and The Hague. Fliers included a QR code to the study website, where potential participants could access full study information and complete an online “self-test” to check their eligibility. Interested participants could then fill in a contact form to be invited for an (online or in-person) intake interview. Recruitment started in August 2020 and ended in September 2022.

#### Intake Interview

During the intake interview, participants received study information and signed written informed consent. The main inclusion and exclusion criteria for the study were then examined with the CSSRS, MINI, and SCID-PD-BPD (see *Participants*). In case the participant was in need of immediate mental health support, they were referred for treatment or crisis management. No participants examined required such immediate intervention.

After meeting eligibility criteria and signing informed consent, and prior to receiving study instructions, a personalized suicide safety plan was created with each participant, detailing available resources and coping strategies available in the event of a suicidal crisis. Participants were also informed that the content of their entries in the EMA app would not be monitored in real time, and in the event of a crisis, the participants should contact their GP and/or treating specialist, or one of the listed support resources (including the suicide prevention line 113). In acute danger situations, participants were instructed to call the emergency number (112). A statement at the end of the safety plan urged participants to immediately contact the study personnel in case they felt that the study proceedings were negatively affecting their mood and/or functioning. No participants reached out to the study personnel to indicate such effects. Participants were also reminded of their right to drop out of the study at any point and without having to provide a reason. Furthermore, the GP and/or treating specialist of all participants was informed of their involvement in the study via a standardized letter.

At the end of the intake, participants received instructions for the EMA. Participants were instructed to download the app (Ethica (a.k.a. Avicenna)), and to enable all notifications necessary for receiving alerts. The researcher then illustrated the use of the app with a demo questionnaire on their phone. Participants were informed they would receive four alerts per day, at pseudo-randomized times (i.e., random times within fixed windows 7:00 am–9:00 am, 12:00 pm–2:00 pm, 4:00 pm–6:00 pm, 8:00 pm–10:00 pm); a reminder alert was sent out 30 minutes after the initial prompt in case participants had not yet filled in the EMA. After the prompt, participants had 180 minutes to fill in the morning assessment, and 120 minutes to fill in the afternoon and evening assessments. The app does not allow participants to save their progress and return to the questionnaire later, but participants were required to fill in the EMA in one go. Likewise, the app does not save partially filled in responses, so only EMA entries that were completely filled in were recorded. Participants could also fill in additional EMA entries at any point in time; the suggestion was that this was something participants could do, for example, to compensate for missed entries (if they wished to do so), or in case they wanted to record specifically high or low moments during their day that were not covered by the scheduled entries. Based on visual examination of the data it appears that participants most often completed additional entries after missing out on a scheduled alert that had recently expired, or filled in additional EMA late in the evening after the final scheduled alert of the day. Even though the content of the participants’ EMA entries was not checked during the data collection period, the research personnel monitored the number of completed/expired surveys through the Ethicadata.com (a.k.a. Avicennaresearch.com) website, and participants received a phone call in case no EMA was completed for 72 hours. The primary purpose of this phone call was to troubleshoot any technical issues with the app; however, in case suicidal crises were encountered, the researchers would follow appropriate steps to direct the participant to contact either their GP/treating specialist, or crisis services. No phone calls required this intervention during the study. Finally, participants received printed instructions for the EMA app (detailing the information covered during the meeting and their login details).

Following the intake interview, participants received an email link to a set of online questionnaires examining additional baseline characteristics (see *Instruments—Questionnaires*) that they were instructed to fill in at home within the 72 hours following the intake interview; participants received a reminder email had they not filled in the questionnaire within the assigned period.

Participants subsequently received an invitation for a post-test meeting organized approximately a week after the end of the EMA period. During this meeting, participants returned the research materials and received instructions for the second phase of the study (as part of the SAFE study participants also underwent 24-hour per day actigraphy over the 3-week EMA period, followed by 1 year of weekly EMA questionnaires; these measures are not included in this article). The researcher also briefly discussed the EMA experience with the participant. In addition, participants were informed during the intake interview that they would receive a personalized feedback report based on their data during the post-test meeting. None of the participants indicated during the intake that they did not wish to receive the report. However, one participant who dropped out during the EMA period, as well as five participants who opted not to continue into the second phase of the study, indicated that they did not wish to attend the post-test session or receive the feedback report. Therefore, 76 participants (93%) received a feedback report. For these participants, during the post-test meeting, the researcher presented them with their personalized feedback report and explained/discussed the report with the participant. Following the meeting, participants received an email with a link to another set of online questionnaires, comprised of the same core set of questionnaires filled in at baseline, with additional items included on the participants’ experience with the EMA. Participants again were instructed to fill in the questionnaire within the following 72 hours and received a reminder email if they did not do so. Participants received a monetary compensation (20€) after completing the 3-week EMA and returning the study materials; compensation was not based on the number of EMA completed. Travel/and or postage costs for study materials were compensated for all participants if applicable.

### Statistical Analysis

All analyses were performed with SPSS. Descriptive statistics were used to present sample characteristics, EMA response rates, and to summarize participant feedback. Linear regression analyses, independent samples *t*-tests and chi-square tests were used to examine predictors and patterns of response rates. Paired samples *t*-tests were used to examine differences between baseline and post-EMA scores on questionnaire measures. Multilevel linear regression analyses (linear-mixed models) were used to assess reactivity in momentary positive and negative affect and suicidal ideation over time. The models included both a random intercept and a random slope, to account for heterogeneity in individual symptom trajectories. A first-order autoregressive (AR) covariance structure was used, which assumes that successive observations are more highly correlated than temporally more distal observations. In line with Husky and colleagues (2014), we used assessment number (1–81) and day number (1–21) as continuous predictors. In the analyses on the effects of assessment number, we specified a three-level structure whereby observations were nested within individuals and within days. In the analyses on the effects of day number, we specified a two-level structure whereby observations were nested within individuals. Finally, we performed post hoc multilevel analyses with the three suicidal ideation items (wish to live, wish to die, suicidal thoughts) as separate outcomes, in accordance with findings that different aspects of suicidal thinking may present different temporal patters (Oakey-Frost et al., 2023). Significance was determined at alpha = .05. With 82 participants and 81 responses per participant as target, and based on the average EMA response rate (78%), we had power (.90) to detect small effects (*d* = .20) (Kleiman et al., 2017).

## Results

### Acceptability

**Figure 1.**
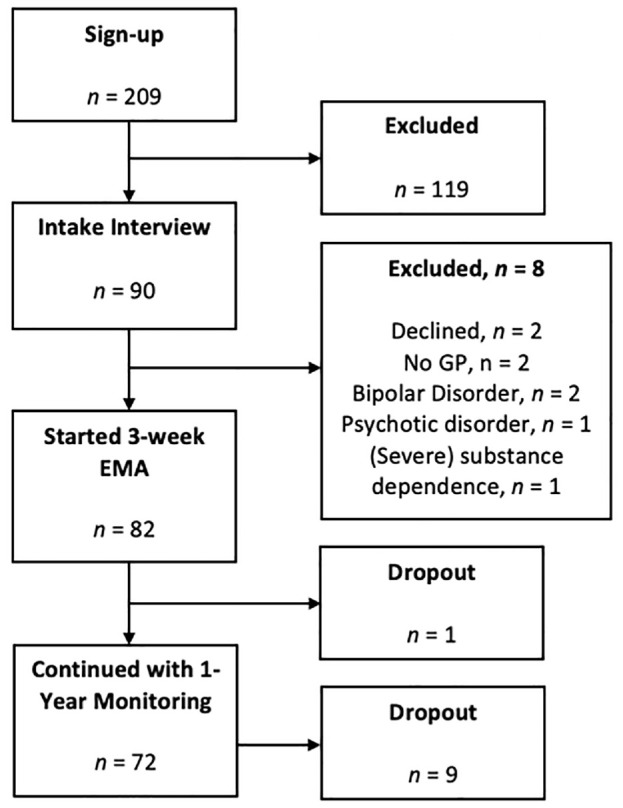
Participant Flow. *Note.* EMA = ecological momentary assessment; GP = general practitioner.

A total of 209 participants signed up for the study and were invited for an intake interview. Of those, 90 attended the intake. Following the interview, eight participants were excluded because they declined to participate (*n* = 2), were not registered with a local GP (*n* = 2), or had probable bipolar disorder (*n* = 2), (primary) psychotic disorder^1^ (*n* = 1), or (severe) substance dependence (*n* = 1). Consequently, 82 participants were enrolled in the study. This resulted in estimates of acceptability ranging from 39% (percentage of participants who signed up for the study and subsequently started the data collection period) to 98% (percentage of eligible participants who completed the intake and subsequently started the data collection period). One participant dropped out of the study during the 3-week EMA period, resulting in a retention rate of 99% (*n.b.* prior to dropping out, this participant achieved a response rate that was within the range of the completers, and hence this participant was retained in all analyses). Participant flow is presented in Figure 1, and an overview of the sociodemographic and clinical composition of the sample is reported in Table 2.

**Table 2.** Sociodemographic and Clinical Characteristics of the Sample.

Sample characteristic	*N* = 82
Gender (*N*, %)	
Female	63 (77%)
Male	11 (13%)
Nonbinary/trans	8 (10%)
Age (*M, SD*)	27 (8.6)
Nationality (*N*, %)	
Dutch	45 (55%)
Other	37 (45%)
Education level (*N*, %)	
Low	11 (13%)
Middle	34 (42%)
High	37 (45%)
Employment (*N*, %)	
Employed	24 (29%)
Not employed	14 (17%)
Student	44 (54%)
Living situation (*N*, %)	
Alone	27 (33%)
With others	53 (65%)
Hospitalized	2 (2%)
Relationship status (*N*, %)	
In a relationship	29 (35%)
Single	53 (65%)
Children (*N*, %)	
Yes	8 (10%)
Current psychiatric diagnosis^a^ (*N*, %)	
MDD	41 (50%)
Other depressive disorders	22 (27%)
Anxiety disorders	47 (57%)
ASD	14 (17%)
ADHD	10 (12%)
Eating disorders	5 (7%)
OCD	7 (9%)
PTSD	18 (22%)
BPD	12 (15%)
Alcohol/substance abuse	7 (9%)
Psychoactive medication (*N*, %)	
Anxiolytics/sedatives	20 (24%)
Stimulants	10 (12%)
Antidepressants	33 (40%)
Current suicidal ideation (BSSI) (*M, SD*)^b^	15.3 (8.6)
Current depressive symptoms (BDI) (*M, SD*)^b^	25.5 (9.6)
Suicide attempt history (*N*, %)	
None	47 (57%)
Single attempt	10 (12%)
Multiple attempts	25 (31%)
Medical diagnosis (*N*, %)	
Yes	35 (43%)
Non-psychoactive medication (*N*, %)	
Yes	26 (32%)
Smoking (tobacco) (*N*, %)	
Yes	35 (43%)

*Note.* Education level: Low = elementary school/vocational education; Middle = secondary school; High = university/applied college education; MDD *=* major depressive disorder; ASD = autism spectrum disorder; ADHD = attention-deficit hyperactivity disorder; OCD = obsessive compulsive disorder; PTSD = posttraumatic stress disorder; BPD = borderline personality disorder.

a All diagnoses are based on current diagnoses derived from the MINI/ SCID-PD-BPD, except for ASD which is based on participant self-report.

b *n* = 71.

Seventy-one participants (87%) also filled in the baseline questionnaire, and 59 participants (72%) filled in the post-test questionnaire. Those who did not fill in the baseline questionnaire were significantly more likely to have a suicide attempt history, χ^2^ (1) = 4.69, *p* = .030, *V* = 0.24, and a diagnosis of attention-deficit hyperactivity disorder (ADHD) χ^2^ (1) = 6.79, *p* = .009, *V* = 0.29. Those who did not fill in the post-test questionnaire were more likely to be male, χ^2^ (2) = 7.45, *p* = .024, *V* = 0.30. Conversely, those with a diagnosis of major depressive disorder (MDD), χ^2^ (1) = 4.27, *p* = .039, *V* = 0.23, were *more* likely to fill in the post-test questionnaire; no other differences were observed on sociodemographic or clinical characteristics.

Following the 3-week EMA period, 72 participants (89%) continued to the second phase of the study (i.e., a 1-year monitoring period with weekly EMA; results not reported here). There were no significant group differences between those who continued and those who did not on either sociodemographic or clinical characteristics (all *p*s > .05).

### Feasibility

Participants on average filled in *M* = 63 (*Med* = 68) EMA entries out of the 81 scheduled alerts, with a mean response rate of 78% (*Med* = 84%) and range from 14 to 81 entries completed (17%–100%). In addition, participants on average filled in *M* = 3 (*Med* = 2) additional entries (range 0–13), resulting in a total of *M* = 66 (*Med* = 70) EMA entries completed per participant overall (range 16–88). In total, *K* = 5,400 unique assessments were completed by the sample as a whole, of which *k* = 5,196 were scheduled entries and *k* = 204 were additional entries initiated by the participants.

**Figure 2.**
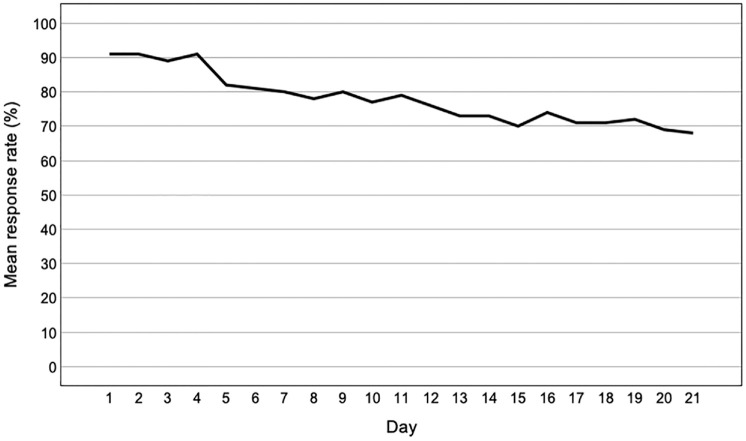
Percentage of Assessments Filled in as a Function of Day Number.

Participants on average filled in the EMA 38 minutes and 21 seconds after the alert, and took 2 minutes and 46 seconds to complete the assessment. The probability of filling in the (scheduled) EMA decreased over time, χ^2^ (1) = 113.37, *p* < .001, OR = 1.06, CI_95%_ [1.05, 1.07], with response rates declining from 91% on Day 1 to 68% on Day 21 (Figure 2). Morning EMA alerts were significantly more likely to be missed, compared to day and evening alerts, with 76% of morning assessments filled in, 79% day and 79% evening, χ^2^ (2) = 10.77, *p* = .005, *V* = 0.04. No differences were observed between weekdays versus weekends (78% response rate on weekdays and 78% on weekends, *p* = .973).

There was no influence of age (*p* = .340), gender (*p* = .127), living situation (*p* = .597), or education level (*p* = .240) on response rates; however, students had lower compliance than non-students (*M_student_* = 74%, *M_other_* = 83%), *t*(79) = 2.12, *p* = .037, *d* = 0.47. There was no influence of borderline personality traits (PAI-BOR, *p* = .056) or suicide attempt history (*p* = .846); however, those with a current diagnosis of an anxiety disorder had lower compliance (*M_anxiety_*= 75%, *M_other_* = 84%), *t*(79) = 2.00, *p* = .049, *d* = 0.45 (all other diagnoses *p* > .05). Baseline quality of life (Q-LES-Q-SR, *p* = .833), depressive symptom (BDI, *p* = .628), suicidal ideation (BSSI, *p* = .223), anxiety (HADS-A, *p* = .302), and insomnia symptom severity (ISI, *p* = .743) also did not impact compliance. However, those scoring higher on trait anger had lower compliance rates (STAXI, *B* = −0.65, *SE* = 0.28, *Beta* = −0.27, *p* = .021).

### Reactivity

**Figure 3.**
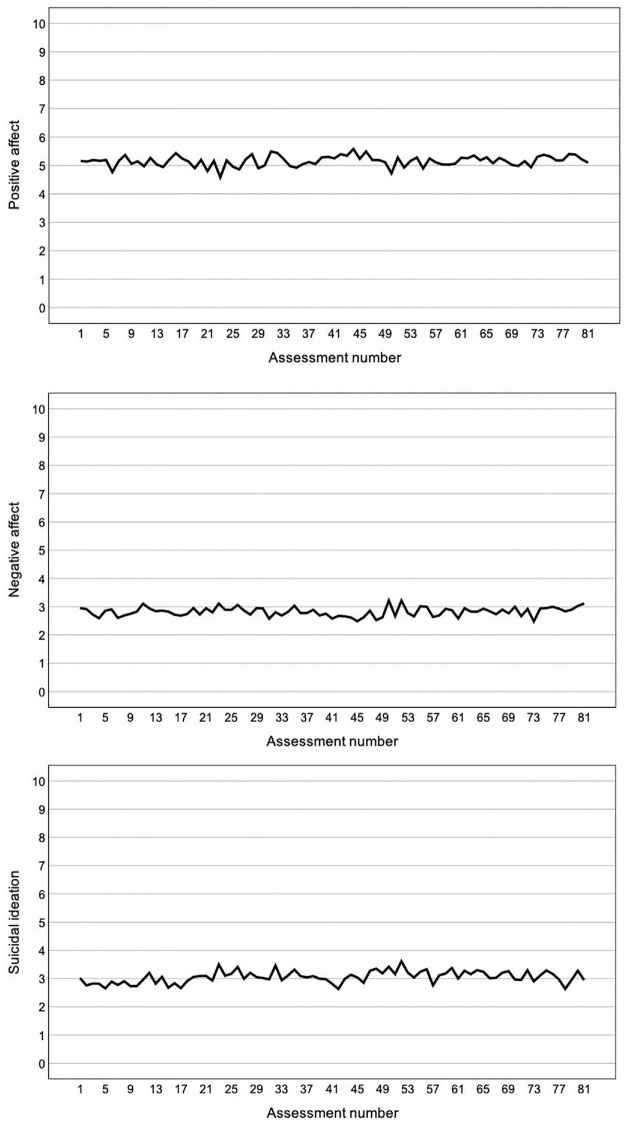
Mean Ratings of Positive Affect, Negative Affect, and Suicidal Ideation as a Function of Assessment Number.

There was no evidence of systematic affect reactivity, that is, increases or decreases in participants’ EMA-rated momentary positive affect (*B* = 0.01, *SE* = 0.09, *p* = .996), negative affect (*B* = 0.01, *SE* = *0.10, p* = .959) or suicidal ideation (*B* = 0.01, *SE* = 0.14, *p* = .973) as a function of assessment number (Figure 3)^2^. Similar findings emerged when examining desire to live (*B* = 0.01, *SE* = 0.16, *p* = .971), desire to die (*B* = 0.01, *SE* = 0.18, *p* = .978), and suicidal thoughts separately (*B* = −0.01, *SE* = 0.12, *p* = .971). There were also no increases or decreases in EMA-rated positive affect (*B* = −0.01, *SE* = 0.08, *p* = .970), negative affect (*B* = 0.02, *SE* = 0.10, *p* = .833), or suicidal ideation (*B* = 0.02, *SE* = 0.14, *p* = .901) as a function of assessment day. Similar findings emerged when examining desire to live (*B* = 0.02, *SE* = 0.16, *p* = .891), desire to die (*B* = 0.02, *SE* = 0.18, *p* = .918), and suicidal thoughts separately (*B* = −0.01, *SE* = 0.12, *p* = .963). Baseline and post-EMA questionnaire comparisons showed a decrease in overall suicidal ideation severity on the BSSI: *M_baseline_* = 16.40 (*SD* = 9.17), *M_post-EMA_* = 15.05 (*SD* = 8.64), *t*(54) = 2.20, *p* = .032, and *d* = 0.30. No differences were observed on the BDI, HADS, ISI, or Q-LES-Q (all *p* values > .05).

### Participant Feedback After 21-Day EMA

Based on participant feedback (*n* = 58; Table 3), the most frequently reported reasons for missing EMA were being otherwise engaged/busy (66%), not having access to phone (20%), and technical issues with the app (20%). Many also reported having missed morning and/or evening assessments due to being asleep (17%).

**Table 3.** Summary of Participant Feedback After the 21-Day EMA Period.

Question	*N* = 58
Overall experience	
Positive	40 (69%)
Neutral	13 (22%)
Negative	5 (9%)
Burdensomeness	
Not burdensome	42 (72%)
Neutral	6 (10%)
Burdensome	10 (17%)
Stressfulness	
Not stressful	43 (74%)
Neutral	9 (16%)
Stressful	6 (10%)
Duration of EMA period	
Just right	48 (83%)
Neutral	2 (3%)
Too long	8 (14%)
Frequency of EMA	
Just right	37 (64%)
Neutral	8 (14%)
Too many	13 (22%)
Number of questions per EMA	
Just right	37 (64%)
Neutral	14 (24%)
Too many	7 (12%)
Number of answer options	
Too few	20 (35%)
Just right	38 (65%)
Too many	—
Reason for missing alerts	
I did not miss any alerts	2 (3%)
Burden too high	9 (15%)
Technical problems	12 (20%)
Too busy	39 (66%)
Phone not accessible/available	12 (20%)
Other	17 (29%)
Change in daily behavior/schedules	
Did not change behavior/schedule	51 (88%)
Neutral	2 (3%)
Changed behavior/schedule	5 (9%)
Improved mood after EMA	
No	36 (62%)
Neutral	9 (16%)
Yes	13 (22%)
Worsened mood after EMA	
No	34 (59%)
Neutral	11 (19%)
Yes	13 (22%)
Triggered suicidal ideation after EMA	
No	35 (61%)
Neutral	12 (21%)
Yes	10 (18%)
Worsened suicidal ideation after EMA	
No	43 (74%)
Neutral	9 (16%)
Yes	6 (10%)

*Note.* EMA = ecological momentary assessment.

Most participants (69%) reported their experience with the EMA as positive overall (22% neutral and 9% negative). About 17% reported the EMA to have been burdensome (10% neutral, 72% not burdensome) and 10% stressful (16% neutral, 74% not stressful); of those who reported the EMA to have been stressful (*n* = 6), two participants indicated the source of the stress to have been the burden of filling in the assessments, one the content of the EMA, and three indicated stress from both the burden and content. In addition, out of a number of descriptive items provided to the participants (selecting multiple items allowed), 48% described the study as “insightful,” 15% “fun/exciting” and 10% “relaxing.” Meanwhile, 12% described the EMA period as “depressing” and 10% “annoying.” The experience for many was multifaceted (e.g., “A lot of work, but also provided insights and sometimes it gave comfort.”).

When asked if participants had changed their daily behavior and/or schedules in some way due to study participation, most (88%) reported no change (3% neutral, 9% changed behavior). Those who indicated (at least some) behavioral change, reported spending more time on their phone (*n* = 3) and waking up earlier so not to miss the morning assessments (*n* = 5), or generally having made positive changes to their sleep (*n* = 1). Ten participants indicated having been more attentive/in tune with their experiences and emotions (“I took more time out of my day to assess how I was feeling.”), of which three indicated having engaged in (positive) behavioral change due to this awareness (“I — was more aware of how bad things were and therefore tried to get into a healthier pattern.” and “I became more aware of my daily rhythms and tried to implement more structure into my days.”).

Most participants reported neither positive mood effects (62% no improvement in mood, 16% neutral, 22% improved mood) nor negative mood effects (59% no worsening of mood, 19% neutral, 22% worsened mood) resulting from the EMA. About 18% reported a triggering effect of the EMA on their suicidal ideation (21% neutral, 61% no triggering effect), and 10% reported a worsening in their suicidal ideation (16% neutral, 74% no worsening effect). Those with more borderline personality traits (PAIBOR, *B* = 0.06, *SE* = 0.02, *Beta* = 0.34, *p* = .013) and those with a posttraumatic stress disorder (PTSD) diagnosis (*B* = 1.17, *SE* = 0.55, *Beta* = 0.28, *p* = .037) were more likely to report a triggering effect of the EMA on their suicidal ideation. Those with higher suicidal ideation (BSSI, *B* = 0.06, *SE* = 0.03, *Beta* = 0.30, *p* = .030), depressive (BDI, *B* = 0.05, *SE* = 0.02, *Beta* = 0.29, *p* = .033), and anxiety symptoms (HADS, *B* = 0.16, *SE* = 0.06, *Beta* = 0.34, *p* = .013), and those with more borderline personality traits (PAIBOR, *B* = 0.05, *SE* = 0.02, *Beta* = 0.28, *p* = .041), were more likely to report suicidal ideation worsening from the EMA; no other participant characteristics were associated with increased suicidal ideation or negative affect reactivity.

When examining the EMA ratings of the subgroup of participants who reported mood worsening (*n* = 13), no increase in negative affect was observed over the EMA period (*B* = 0.01, *SE* = 0.26, *p* = .967). When examining the EMA scores of the subgroup of participants who reported triggering (*n* = 10) or worsening of suicidal ideation (*n* = 6), no increase in suicidal ideation was observed over the EMA period (triggering: *B* = −0.02, *SE* = 0.35, *p* = .958; worsening: *B* = −0.01, *SE* = 0.53, *p* = .994). Notably, all participants who filled in the feedback survey (including those who reported iatrogenic effects) continued into the second phase of the study.

## Discussion

In this study, we examined the acceptability and feasibility of EMA in patients with suicidal ideation, with a focus on iatrogenic effects and identifying subgroups of patients who may be more affected by negative reactivity. Overall, our findings support the acceptability, feasibility, and safety of EMA among patients with current suicidal ideation. While we failed to uncover systematic iatrogenic effects in EMA-rated affect and suicidal ideation, a distinctive subgroup of participants (characterized by higher depression, anxiety, and suicidal ideation severity, as well as comorbid PTSD and BPD traits) self-reported experiencing negative reactivity from the EMA, based on participant feedback after the 21-day EMA period. These findings are discussed further below.

### Acceptability

With 39% of those signing up for the study ultimately starting the EMA, our acceptability rate was fairly low. Online-based recruitment is likely to attract a higher number of people curious about the study rather than serious intent to participate. Studies approaching potential participants in inpatient or outpatient settings tend to report higher acceptability rates (see e.g., Husky et al., 2014; Torous et al., 2015). Meanwhile, 98% of participants who attended the intake interview and were deemed eligible to participate started the EMA period. Our 99% retention rate was also higher than that reported in the literature (60%–96%) (Czyz et al., 2018; Forkmann et al., 2018; Law et al., 2015; Porras-Segovia et al., 2020; Rogers, 2021). These numbers are likely influenced by participant self-selection; those following up with the intake interview were likely to have already carefully considered the burden of participation and were more intrinsically motivated to take part in the study.

### Feasibility

We achieved excellent compliance rates, with people on average filling in 78% (*Med* = 84%) of the scheduled EMAs. As such, our compliance rate was higher than the average in previous studies (*Med* = 70%) (Kivelä et al., 2022). Reasons for our high compliance are again likely to include participant characteristics and self-selection, as well as the nature of the incentives used in the study; participants were aware that they would receive a personalized feedback report which was dependent on the (amount and quality) of their EMA responses. Notably, we did not employ additional feedback or rewards for increased compliance, such as periodically providing participants with feedback on their response rate, or offering additional monetary rewards for high compliance (as done previously by, for example, Glenn et al., 2020; Rogers, 2021). Indeed, monetary rewards tend to have fairly small effects on compliance (Ottenstein & Werner, 2021), whereas more personalized rewards (such as feedback reports) may be more effective in increasing participants’ engagement with the study (Folkersma et al., 2021). Participants were also informed they would receive a phone call from the study personnel if they did not fill in any EMA for 72 hours; desire to avoid this phone call may have further increased participants’ compliance. However, our decision not to monitor the *content* of participants’ responses in real time may also have influenced responses and response patterns: while response monitoring is generally recommended (especially when studying adolescents) it is also understood that such monitoring may lead to underreporting of suicidal ideation, or even additional missing data in case participants stop completing the surveys at times of severe ideation to prevent unwanted intervention by research staff (Bentley et al., 2021).

While previous studies have concluded that participant characteristics, such as suicide attempt history or current depression or suicidal ideation severity, do not influence response rates (Glenn et al., 2020; Hallard et al., 2021; Oquendo et al., 2020; Peters et al., 2020; Rogers, 2021), we identified several characteristics that were predictive of lower compliance. Our finding that students had lower compliance than non-students is contrary to Porras-Segovia and colleagues (2020), who reported higher compliance among student controls than psychiatric patients. However, most of our student participants also had current psychiatric diagnoses, therefore hindering direct comparisons with the previous study. Furthermore, we also found lower compliance among those with an anxiety disorder, as well as those scoring higher on trait anger. Lower compliance among patients with anxiety disorders may be explained by anxious individuals’ propensity to experiential avoidance (i.e., avoidance of distressing emotional experiences) (Hayes-Skelton & Eustis, 2020), which may have reduced their willingness to attend to their internal states as prompted by the EMA. Meanwhile, trait anger is correlated with both low agreeableness and low conscientiousness (Pease & Lewis, 2015), which can logically be expected to also extend to lower study compliance.

It is more difficult to infer how our study design may have impacted compliance. At 21 days, our assessment period was fairly long (average study duration in previous studies *Med* = 14), while the number of assessments per day (4) was slightly below average (*Med* = 5) (Kivelä et al., 2022). However, with up to 40 questions per EMA prompt our protocol was fairly intensive. Most previous studies achieving comparable compliance rates (>70%) employed shorter assessment periods (≤2 weeks) (Husky et al., 2017; Littlewood et al., 2019; Nock et al., 2009; Oquendo et al., 2020; Spangenberg et al., 2019) or only collected EMA once per day (Coppersmith et al., 2019; Czyz et al., 2020). However, Victor and colleagues (2019) reached similar compliance in an EMA study of young women with a history of self-injurious thoughts, which employed seven daily prompts over 21 days. Finally, unlike many other studies (Glenn et al., 2020; Kleiman et al., 2017; Littlewood et al., 2019; Rizk et al., 2019) that allowed participants to adjust the EMA prompt windows to their daily schedules (e.g., wake up and bedtimes), we employed the same assessment schedule for all (7:00 am–10:00 pm), to create comparable timeframes between participants that would allow us to examine time-of-day effects in future analyses. However, to provide the participants with some additional flexibility in terms of their response times, we allowed for a time window of 3 hours in the mornings, and 2 hours during the daytime and evenings, for the participants to complete the EMA following the initial alert. Regardless, this may have led to the lower compliance we observed to morning assessments (with non-morning types being more likely to miss early alerts), although it has also previously been reported that adherence to morning surveys tends to be lower than that to daytime assessments (Jacobucci et al., 2023; Torous et al., 2015). We also experienced decreasing compliance over time, with compliance rates declining from 91% to 68% between the first and last day of the assessment period, indicating some fatigue effects. Decreasing compliance with increasing study duration is a consistent finding in the literature (Czyz et al., 2018; Forkmann et al., 2018; Glenn et al., 2020), with a distinctive drop after 3 weeks (Jacobucci et al., 2023). For example, in a study by Czyz and colleagues (2018), compliance decreased from 80% on week 1 to 60% on week 4, and in a study by Glenn and colleagues (2020) from 87% on week 1 to 45% on week 4. Notably, both previous studies used adolescent samples.

Of note is also that we experienced some technical issues with the EMA app several times over the 26 months of data collection but unfortunately were unable to account for the exact amount of missing data that was due to technical issues (rather than noncompliance). However, 20% of participants reported having been impacted by technical issues; some also reported that frustration with the technical issues reduced their engagement with the study and therefore lead to additional missed entries.

### Reactivity

Importantly, no suicide attempts or deaths occurred during the EMA period. Examination of changes in participants’ EMA-reported positive and negative affect and suicidal ideation over the study period indicated no (negative or positive) affect reactivity. This is in line with prior studies showing no increases in negative affect, suicidal ideation, or other suicide outcomes in response to EMA measures (Coppersmith et al., 2022; Husky et al., 2014; Law et al., 2015). While these prior studies showed no reactivity in active suicidal ideation (thoughts about, and desire and intent for suicide), we also considered more passive aspects of ideation (desire to live, desire to die), which neither exhibited reactive effects. However, 22% of participants retrospectively indicated having experienced mood worsening during the study period, with 18% of participants having experienced the EMA as triggering their suicidal ideation and 10% as worsening their ideation. These numbers seem to largely align with previous studies: 16% of depressed inpatients reported having experienced EMA as stressful and/or burdensome (Forkmann et al., 2018), and 9% of a community-based sample with current suicidal ideation stated the assessments to have been “occasionally ‘distressing,’ ‘emotionally taxing’ or ‘triggering bad thoughts’” (Rogers, 2021). When examining the characteristics of those who were more likely to report iatrogenic effects, we found increased symptom severity (depression, anxiety, and suicidal ideation), as well as comorbid PTSD and BPD traits, to distinguish those who were more likely to report reactivity. Individuals experiencing more severe current symptoms may find the study proceedings as more taxing or more confrontational, due to the higher number of negative emotional experiences they would be forced to face. Individuals with BPD traits specifically (Sansone & Sansone, 2010; Sauer et al., 2014), as well as those with PTSD (Badour & Feldner, 2013; Sauer et al., 2014), are also more likely to experience problems with emotion regulation, including emotional (hyper)reactivity. Furthermore, this emotional (hyper)reactivity does not only concern negative but may even result from neutral environmental stimuli (Sansone & Sansone, 2010). Individuals higher in BPD traits are also less likely to engage in emotional acceptance (Chapman et al., 2013) and may hence experience their emotions as more distressing. Meanwhile, an EMA study showed avoidance to be the most frequently used emotion regulation strategy by patients with PTSD and that maladaptive emotion regulation prospectively predicted increases in PTSD symptoms (Short et al., 2018). Consequently, patients with PTSD may be more distressed by facing their (negative) emotions.

It should also be noted that the participants’ self-report with regard to these iatrogenic effects was completed, on average, 1 to 2 weeks after the end of the EMA period and concerned the assessment period as a whole, and we did not include questions as part of the EMA itself to inquire whether participants felt iatrogenic effects *in the moment*. As such, it is impossible to assess if participants experienced this subjective reactivity in *real time*, and these reports may further be influenced by retrospective memory biases. For example, an EMA study on PTSD symptoms concluded that retrospective symptom reports post-EMA more closely corresponded to worst-point EMA scores, rather than average ratings throughout the EMA period (Schuler et al., 2021). Patients with depression are also known to exhibit negative memory biases, with the strength of such biases being associated with symptom severity (Duyser et al., 2020). Individuals with borderline personality traits also have a tendency to recall negative experiences in a manner where the reported severity of the experience increases over time (Maraz et al., 2022). We also did not ask whether participants experienced *decreased* suicidal ideation after filling in EMA, so our questionnaire was biased toward participants reporting more negative rather than positive reactive effects. Furthermore, all participants who filled in the feedback survey (including those reporting iatrogenic effects) continued into the second phase of the study. As part of their safety plan, participants were also urged to immediately contact the study personnel in case they felt that the study proceedings were negatively affecting their mood and/or functioning; none of the participants made contact for this reason. Hence, in concordance with our findings of no systematic reactivity in the participants’ EMA scores, it appears that for those reporting iatrogenic effects the negative reactivity was unlikely to have been systematic, or substantially distressing. In line with participant reports that they experienced the EMA as increasing their awareness of their emotions and daily experiences (e.g., “I — was more aware of how bad things were and therefore tried to get into a healthier pattern.”), it may be that, for better or worse, this increased attention and awareness may also have led to increased focus on negative emotions. Hence, the EMA may have forced some participants to confront emotions they were trying to ignore or suppress, resulting in temporary mood and/or suicidal ideation worsening after filling in the assessments. Alternatively, these reports may simply reflect participants’ increased attention to their thoughts and emotions that were already there (including suicidal ideation), rather than actual increases in the intensity of said experiences. As EMA has been shown to increase emotional self-awareness (Kauer et al., 2012), this awareness might be perceived as the triggering or worsening of suicidal ideation by EMA. Correspondingly, prior research has demonstrated that neither suicidal ideation (Coppersmith et al., 2019; Husky et al., 2014) nor suicidal behavior (Law et al., 2015) increase in response to EMA. Other participants also reported that having to fill in certain responses, such as repeatedly reporting that they were alone when filling in the EMA, sometimes made them feel sad, illustrating how even innocuous questions may sometimes be triggering. A further point of consideration that has recently been brought forward as explaining effects that may appear iatrogenic concerns the emotion regulation function of suicidal thinking (Coppersmith et al., 2023; Kleiman et al., 2018). This emotion regulation function may explain why certain participants (i.e., those using suicidal thinking as a form of maladaptive coping) may experience increases in suicidal thinking over time. This is based on findings that those who report engaging in suicidal thinking as a form of emotion regulation are more likely to report more frequent and severe suicidal thoughts (Coppersmith et al., 2023).

Finally, we observed a decrease in overall suicidal ideation severity from baseline to post-EMA (on the BSSI). This finding is contrary to our findings of no systematic change in the participants EMA-rated suicidal ideation. To the best of our knowledge, no previous EMA study has reported decreases in suicidal ideation following study participation. However, studies employing other cross-sectional and longitudinal designs have shown that participating in suicide research may serve to lessen suicidal ideation (Schatten et al., 2022; Smith et al., 2010). However, our finding of reduced suicidal ideation on the BSSI is likely to also be influenced by the lower compliance to the post-test questionnaire (71%), with those in a better mental state perhaps being more willing to fill in the additional assessment. An alternative explanation concerns potential intervention effects resulting from the feedback reports presented to the participants after their EMA period (and prior to filling in the post-EMA questionnaire, which included feedback about the study). It is possible that, rather than the EMA procedure itself, the insights resulting from the feedback report and related discussions with the research personnel may have led to symptom relief. Unfortunately, we did not formally evaluate the participants’ reactions to the feedback reports, as the study was designed as an observational rather than an intervention study, and the feedback reports were merely intended as additional incentives for participants, and neither the EMA assessments nor the feedback reports were expected to lead to treatment effects. However, with 22% of participants reporting *improved* mood in response to the EMA, it is clear that reactive effects may also appear in a positive direction.

Strengths of our study include a diverse high-risk sample, as we employed minimal exclusion criteria related to comorbidities, medication use, and so on. As such, our findings have greater generalizability to the heterogeneous group of patients experiencing suicidal ideation. Furthermore, as we achieved higher retention and compliance rates than expected, we had excellent power for our analyses. Finally, we paid special attention not only to objective iatrogenic effects, but participants’ subjective experiences in undergoing intensive longitudinal assessments on suicidal ideation.

Limitations of this study include the relatively small sample; although our sample size is somewhat higher than the average in past studies (*Med* = 52) (Kivelä et al., 2022), larger-scale studies are needed to replicate these early findings. Furthermore, although we achieved excellent compliance with the EMA, compliance with other study proceedings (such as the baseline and post-test questionnaires) was lower. Hence, the subsample of participants who reported on their experience with the EMA may not be representative of the full sample, and most importantly may neglect to take into account those who experienced more substantive negative effects. Finally, the exclusion of participants with current bipolar, psychotic, or severe substance abuse disorders limits the generalizability of our results when considering patients with the aforementioned comorbidities.

In conclusion, high feasibility numbers should not blind researchers to the fact that a distinctive minority may report negative reactivity in response to repeated daily assessments of suicidal ideation. These retrospective reports did not, however, correspond with changes in momentary mood and/or suicidal ideation during the EMA. Regardless, increased attention in future research should be paid to identifying subgroups of patients who may be more likely to report negative effects. Based on our findings, this may include those with higher baseline symptom severity (depression, anxiety, and suicidal ideation) as well as comorbidity with either PTSD or BPD traits. Participants in similar studies should be transparently informed that they may experience mood effects—whether those be positive or negative.

## Supplemental Material

sj-docx-1-asm-10.1177_10731911231216053 – Supplemental material for Examination of Acceptability, Feasibility, and Iatrogenic Effects of Ecological Momentary Assessment (EMA) of Suicidal Ideation Supplemental material, sj-docx-1-asm-10.1177_10731911231216053 for Examination of Acceptability, Feasibility, and Iatrogenic Effects of Ecological Momentary Assessment (EMA) of Suicidal Ideation by L. M. M. Kivelä, F. Fiß, W. van der Does and N. Antypa in Assessment

## Notes

1. Participants with major depressive disorder (MDD) with psychotic features were included. Participants with primary psychotic disorders (as per *DSM-5* definition) such as schizophrenia were excluded.

2. Analyses on response rates and reactivity were based on scheduled alerts only in order to keep the number as well as timing of the entries consistent across participants.
