# Supplementary material for: Examination of Acceptability, Feasibility, and Iatrogenic Effects of Ecological Momentary Assessment (EMA) of Suicidal Ideation
Source: Assessment. 2023 Dec 14;31(6):1292–308. doi: 10.1177/10731911231216053 (PMC11292966; doi:10.1177/10731911231216053)
Supplement: sj-docx-1-asm-10.1177_10731911231216053 – Supplemental material for Examination of Acceptability, Feasibility, and Iatrogenic Effects of Ecological Momentary Assessment (EMA) of Suicidal Ideation [file sj-docx-1-asm-10.1177_10731911231216053.docx]

**Supplementary material**

Daily EMA questions (21-day assessment)

SLEEP**

Good morning!

1. How did you sleep last night? From 0 (very poorly) to 10 (very well).
2. What time did you go to bed? _____
3. What time did you try to get to sleep? _____
4. How long did it take you to fall asleep? _____
5. Did you wake up during the night? (if YES, go to A; if NO, go to 6)
   1. How long were you awake (in minutes)? _____
6. Did you have any nightmares? _____
7. What time did you wake up for the day? _____
8. What time did you get out of bed? _____
9. Did you take any naps today? (if YES, go to A; if NO, go to ADDITIONAL COMMENTS)***
   1. How many minutes in total did you spend napping?

ADDITIONAL COMMENTS

1. Do you want to add any other comments/notes about your sleep? _______

****Only assessed at the first EMA beep of the day.**

*****Only assessed at the last EMA beep of the day.**

CONTEXTUAL FACTORS (adapted from (Husky et al., 2017))

1. Where are you right now? (Select: at home; work; other:_____)
2. What are you doing right now? _____
3. Currently I am… (Select: alone, with others)

If “with others”, Select: friends; family; other:_____

IMPACTFUL EVENTS (adapted from (Chaudhury et al., 2017))

1. Have you experienced any events* that had an impact on you since the last questionnaire? (if YES, go to A; if NO, go to SUBSTANCE USE)
   1. Please indicate the type of event that had the most impact: (Select: had a disagreement with someone; been rejected by someone; been complimented or praised by someone; been disappointed by someone; felt neglected by someone; experienced a loss of some sort; received good news; received bad news; been reminded of something painful from the past; been reminded of something pleasant from the past; other: something negative_______; other: something positive_______)
   2. How stressful was the (most stressful) event? From 0 (not stressful at all) to 10 (very stressful).

*These events may be either negative *or* positive.

MOOD (adapted from (Husky et al., 2017; Links et al., 2007))

1. At the moment, how *happy* do you feel? From 0 (not at all) to 10 (very much) (Positive mood)
2. At the moment, how *calm* do you feel? From 0 (not at all) to 10 (very much) (Positive mood)
3. At the moment, how *sad* do you feel? From 0 (not at all) to 10 (very much) (Negative mood)
4. At the moment, how *anxious* do you feel? From 0 (not at all) to 10 (very much) (Negative mood)
5. At the moment, how *angry* do you feel? From 0 (not at all) to 10 (very much) (Negative mood)
6. At the moment, how *guilty* do you feel? From 0 (not at all) to 10 (very much) (Negative mood)
7. At the moment, how *ashamed* do you feel? From 0 (not at all) to 10 (very much) (Negative mood)

COGNITIONS (adapted from (Kleiman et al., 2017))

1. At the moment, how *hopeless* do you feel? From 0 (not at all) to 10 (very much).
2. At the moment, how *optimistic* do you feel? From 0 (not at all) to 10 (very much).
3. At the moment, how *lonely* do you feel? From 0 (not at all) to 10 (very much).
4. At the moment, I feel like *I’m a burden to others in my life*. From 0 (not at all) to 10 (very much).

SUICIDAL IDEATION (adapted from (Kleiman et al., 2017; Ribeiro et al., 2014))

1. At the moment, how strong is your desire to live? From 0 (none) to 10 (very strong) (Passive ideation).
2. At the moment, how strong is your desire to die, or to go to sleep and not wake up? From 0 (none) to 10 (very strong) (Passive ideation).
3. At the moment, do you actually have thoughts of killing yourself? From 0 (not at all) to 10 (very much) (Active ideation). (If ≥ 1, go to A; if = 0, go to COPING STRATEGIES)
   1. At the moment, how strong is your intention to act on these thoughts? From 0 (none) to 10 (very strong) (Active ideation).
   2. At the moment, how much can you resist the urge to kill yourself? From 0 (not at all) to 10 (very much)? (Acquired capability).
   3. At the moment, how afraid are you of dying? From 0 (not at all) to 10 (very much) (Acquired capability).
   4. At the moment, how afraid are you of the *pain* associated with dying? From 0 (not at all) to 10 (very much) (Acquired capability).

COPING STRATEGIES (adapted from (Chaudhury et al., 2017))

1. If you have experienced negative mood/thoughts, did you do something to try to manage them? (If YES, go to A; if NO, go to SUBSTANCE USE)
   1. What did you do? (Select: keeping busy; socializing; calling/messaging a friend; calling/messaging a family member; positive thinking; doing something good for self; calming self/relaxation; finding perspective; sitting with feelings until they pass; other: _______)

SUBSTANCE USE

Since the last questionnaire have you used:

1. Medication (other than your daily prescriptions)? _______
2. Alcohol? _______
3. Cannabis? _______
4. Other drugs? ______

ADDITIONAL COMMENTS

1. Do you want to add any other comments/notes? _______

Experience with EMA questionnaire

The following questions are about your experience measuring your mood / thoughts using the mobile phone (Ethica) app during the past three weeks.

1. How burdensome did you find the mobile phone assessments overall? 0 (not at all) to 7 (very much)
2. The duration of the study (3 weeks) was… 0 (just right) to 7 (too long)
3. The number of assessments per day (4) was… 0 (just right) to 7 (too many)
4. The number of questions per assessment was… 0 (just right) to 7 (too many)
5. Were there specific questions you found difficult or annoying to answer? (text entry)
6. Were there specific questions that you hope would have been included? (text entry)
7. How did you find the answer options / rating scales? 1 (There was always a suitable answer option available), 2 (There were not enough options / the scale was too limited), 3 (There were too many options / the scale was too broad)
8. If you missed assessments during the 3 weeks, did you miss them due to... 1 (I didn't miss any assessments), 2 (The burden of the assessments was too high), 3 (Technical problems / I didn't receive the alert), 4 (I was too busy / I didn't have time), 5 (I didn't have my mobile phone with me), 6 (Other – text entry) (you may choose multiple)
9. To what extent did you change your behavior / normal daily rhythms due to the assessments? 1 (not at all) to 7 (very much)
   1. If yes, how did your behavior / daily rhythms change? (text entry)
10. How stressful was filling in the assessments? 1 (not at all) to 7 (very much)
    1. What part of the assessment did you find stressful? 1 (The process of filling in the assessments (i.e., time burden, missing assessments, difficulty using the app etc.)), 2 (The content of the questions (i.e., sensitive topics)), 3 (Both the process and content of the assessments.)
11. Do you think the assessments sometimes influenced your mood in a positive way (i.e., improved your mood / felt better after filling in the assessment)? 1 (not at all) to 7 (very much)
12. Do you think the assessments sometimes influenced your mood in a negative way (i.e., worsened your mood / felt worse after filling in the assessment)? 1 (not at all) to 7 (very much)
13. Do you think the assessments sometimes triggered suicidal thoughts (when you didn't have these thoughts prior to filling in the assessment)? 1 (not at all) to 7 (very much)
14. Do you think the assessments sometimes worsened your suicidal thoughts (when you already had these thoughts prior to the assessment)? 1 (not at all) to 7 (very much)
15. Would you describe your experience with using the app / filling in the assessments as ... ? 1 (Fun/exciting), 2 (Relaxing), 3 (Insightful), 4 (Neutral), 5 (Depressing), 6 (Annoying), 7 (Stressful), 8 (Other – text entry) (you may choose multiple)
16. How would you rate your experience with the mobile phone app assessments overall? 1 (very positive) to 7 (very negative)
17. Would you like to add any other comments? (text entry)
